# Correlates of gender characteristics, health and empowerment of women in Ethiopia

**DOI:** 10.1186/s12905-015-0273-3

**Published:** 2015-12-07

**Authors:** Yishak Abraham Lailulo, A Sathiya Susuman, Renette Blignaut

**Affiliations:** Department of Statistics & Population Studies, University of the Western Cape, Cape Town, South Africa

**Keywords:** Women empowerment, Health seeking behaviour, Occupation, Early child marriage, Wealth index, Family planning

## Abstract

**Background:**

The low status of women prevents them from recognizing and voicing their concerns about health needs. This study aimed to examine the relationship between gender characteristics, health and empowerment of women in an attempt to understand between 2005 and 2011.

**Methods:**

Data from the Ethiopia Demographic and Health Survey (EDHS) 2005 and 2011 were used. Bivariate and multivariate analyses were used to determine the relative contribution of the predictor variables. The hypotheses tested in this study were that gender (men and women), health and empowerment of women in region are highly significant with women’s education and work status.

**Results:**

Study findings showed that the low status of women and their disempowerment are highly associated with poor health outcomes. In both 2005 and 2011 men school ages were positively associated with their attainment in primary education, whereas for women it was negatively related with their attainment in some education. In both 2005 and 2011 women in the richest wealth quintile had the highest odds ratio of relating to some education. The results show that the odds ratios of women with some education (within the richest wealth quintile) has improved from 6.39 (in 2005) to 10.90 (in 2011), whereas among men there has been a decrease from 10.33 (in 2005) to 2.13 (in 2011). The results indicated that in 2005 and 2011, when comparing the percentage distribution of both genders on employment status and type of occupation, the percentage of men who were employed was higher than women. The percentage of males who were engaged in the agricultural-type of occupation was higher than that of women. Men and women knowledge about family planning methods have been improved, yet, there are wider gender gaps in family planning users.

**Conclusions:**

The officials such as policy makers, planners, program managers and government and non-government organizations need to addressed. The issue of child marriages in order to minimize the number of girls who never attend school or drop out to become wives Planners should also work on improving family planning to empower women. There was a significant relationship between status of women and quality of healthy life, and this relationship appeared to differ by education and work status.

## Background

Empowering women defined gender as a set of characteristics, roles, and behaviour patterns that differentiate women from men socially and culturally and relations of power between them [1]. These characteristics, roles, behaviour patterns and power relations continuously change; they differ over time and between different cultural groups because of the constant shifting and variation of cultural and subjective meanings of gender [2]. There are three components in the identification of different roles of men and women: action, locus, visualization and power, among other things [3]. Action refers to sexual division of labour. Actions are generally categorized into three: productive, reproductive, and community activities. Locus shows the environment in which men and women function. It is essential in identifying gender gaps, mainly working at home or away from home. Visualization involves being seen (i.e. acknowledged) and rewarded (both materially and also by privilege) for carrying out certain activities [3]. Several studies have shown that there is generally a low status of women in developing countries, particularly in Ethiopia [2, 4]. In Ethiopia, women generally have a low socioeconomic status which is attributed to a lack of access to essential resources (such as land, education, employment, health services, as well as protection of their rights); in addition to these, women also have a lowered autonomy in that they have low decision making power, and are surrounded by violence and harmful traditional practices [5]. Gender differences in power and roles have an effect on health, fertility control, survival and nutrition of women (thus contributing to a low status of women), and this low status lowers women’s rights over their bodies and sexuality, and is a source of constraint in material and non-material resources [5, 6].

The process of improving gender disparities in a society will result in the empowerment of women and will give them more autonomy in the household as well as at community level. Women’s status can be defined as the level at which women have access to and control over material resources and social resources within the household, in the community, and the overall society [5, 6]. Women’s status is a concept which signifies their autonomy to control essential aspects of their lives, such as control over resources, and ability to make decisions about her life. It can be examined based on different indicators (through the quantitative technique). Variables such as education and employment have been commonly used predictors of women to empowerment in conjunction with other related notions such as women’s status and autonomy; the use of these variables are warranted by the fact that they have an association with the direct indicators relating to women’s empowerment [5]. Maternal education, employment status, as well as media exposure are usually found to be correlated with empowerment [5, 7]. Maternal education increases women’s autonomy in that it opens their minds to their rights as well as to information.

### Ethiopian perspective

Ethiopia is a male-dominated society which automatically puts women in a lower status position [8]. In male-dominated societies, mainly in Africa, there are an acceptance that women should be subservient and tolerant of their partners or husbands (whereby if women go against these norms, then violence is instigated against those who out of line) – and this is all done in the name of culture [2]. Ethiopian societies are socialized in a way whereby women have a lower social status compared to men. From a young age, autonomy is promoted among boys, while girls are not given any autonomy, but they are trained to be more obedient and dependent, and to household chores such as cooking, in preparation for marriage and caring for their households in later years [2, 8]. Low educational level is one of the key causes and consequences of females’ low socio-economic status. Lack of (or low) educational attainment is among one of the major roots of women’s lowered socio-economic status in Ethiopia. Women’s education is usually lower than that of men [5] and until this gap is bridged, it will be difficult for women to be empowered. One of the major reasons that women (or young girls) are not empowered through higher levels of education is the issue of early marriages [5, 9]. Children (young girls) are married off and this has some serious implications on their development and empowerment [9]. Thus, lack of education, other factors contributing to low socio-economic status of the women include lack of access to resources (i.e. land), and lack of access to employment (and financial service) [10]. This is even more evident when it comes to how resources are owned and shared in rural areas throughout Africa – where women have limited decision-makers to empower related to these resources, particularly when it comes to farming and management of natural resources [10]. A study looking into associations between women’s autonomy and reproductive healthcare seeking behaviour found that factors such as maternal education, place of residence, wealth status, employment, and media exposure increases women’s decision-making autonomy [11]. Women’s age is also a major factor when it comes to decision-making about choices regarding sexual intercourse.

A study among Ghana women found that women aged 35 and above were more likely to have autonomy when it came to engaging in sexual intercourse compared with women aged 15–24 [12]. As expected, this same study found that maternal education increases women’s decision-making power regarding reproductive health, particularly condom use – where women with primary and secondary education had higher odds of decision-making power than those who had no formal education [12]. This could be explained by that younger women (i.e. those aged 15–24) tend to be inexperienced when it comes to sexual and reproductive health, which puts them at the most risk of being more agreeable towards their partner’s sexual demands in fears of losing their partners. This study aimed to examine the relationship between empowerment of women and health in an attempt to understand these changes between the two periods (2005 and 2011).

## Methods

**Fig. 1:**
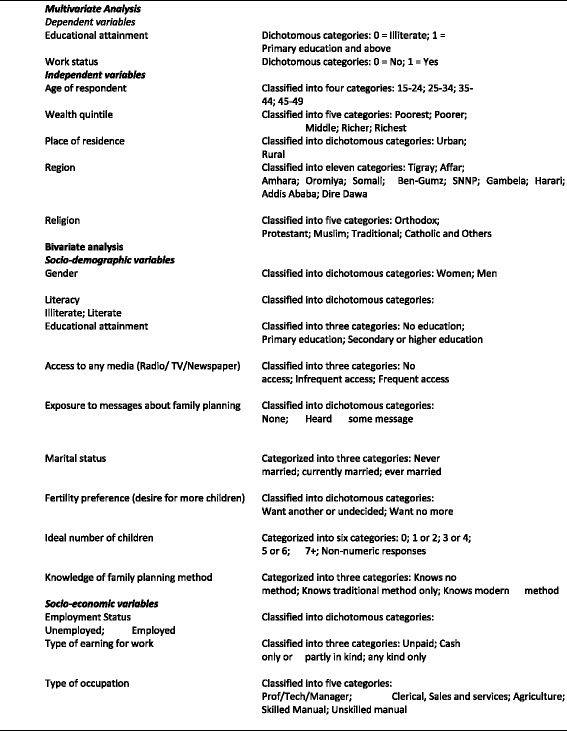
Definition of selected variables

In order to clearly show gender inequality and women’s empowerment in the country, bivariate and multivariate techniques were used based on the variables supplied in both Ethiopian Demographic and Health Survey (EDHS) 2005 and 2011. EDHS, 2005, total number of 14070 eligible women: the study sample 8914 (married women). According to the EDHS, 2011, there were 16515 eligible women but in the study sample used 9594 (married women). Bivariate techniques were used to demonstrate whether there is a significant difference between men and women with respect to selected socio-economic and demographic variables. Multivariate methods were used to determine the relative importance of identified explanatory variables in affecting major indicators for gender gap such as education and employment taken as dependent variables. The main explanatory variables include household characteristics and individual characteristics. Binary regression models were applied for the multivariate analysis. The logistic models I & II consider the association between a binary dependent variable and a set of selected independent variables. There were two models applied, the dependent variables being educational attainment (illiterate = 0; primary education and above =1) and work status (No = 0; Yes = 1) (Fig. 1).

### Ethical considerations

This study used secondary data from the Ethiopian Demographic and Health Survey. Prior using these data, agreement was obtained from Macro International, which has allowed us to download the data from their Web Site. Please note that all data are fully available without restriction.

## Results

The results (as shown in Table 1) highlight a 20 % decline in illiteracy rates among women from 2005 to 2011, where the percentages for illiteracy were 76.8 % in 2005 and 61.6 % in 2011. On the other hand, the literacy rates showed a 79.1 % increase from 2005 to 2011, where the percentages in women’s literacy rates were 21.5 % and 38.5 % between the respective years. These figures represent a remarkable increase in women’s literacy rates. The results show that the male illiteracy rates dropped from 53.3 % (in 2005) to 34.8 % (in 2011), but literacy rates increased from 45 % to 65.3 % in 2011. The percentage of women who do not attend school has declined from 66 % (in 2005) to 50 % (in 2011) – showing a 24 % decrease between 2005 and 2011. The results shown a decrease among men with no education, from 43 % in 2005 to 31.5 % in 2011. Primary education, secondary and higher education among women and men has shown an increase from 2005 to 2011. Among women, secondary and higher education increased by 21 % from 2005 to 2011; whereas among men there was a 7.1 % percentage increase between these two years. The percentage of women and men with no access to any media (Radio, TV/Newspaper) has increased from 2005 to 2011 from 53.7 % to 60.1 % (among women) and 33 % to 40.4 % (among men). Conversely, the percentage of women and men who have heard some messages about family planning on the media have declined from 31.3 % to 23 % (among women) and 40.5 % to 34.8 % (among men) from 2005 to 2011.

**Table 1:** Gender differentials based on selected background characteristics in Ethiopia, DHS 2005 and 2011

Background characteristics	DHS 2005		DHS 2011	
	Women	Men	Women	Men
*Literacy*				
Illiterate	78.80	55.00	61.60	34.80
Literate	21.50	45.00	38.50	65.30
*Educational Attainment*				
No education	65.90	42.90	50.10	31.50
Primary	22.20	37.30	35.50	47.30
Secondary and higher	11.90	19.80	14.40	21.20
*Access to any media (Radio/ TV/Newspaper)*				
No access	53.70	33.00	60.13	40.40
Infrequent access	25.80	30.80	23.27	42.30
Frequent access	20.50	36.20	16.50	18.20
*Exposure to messages about family planning (Radio/TV/News Paper)*				
None	68.70	59.50	77.00	65.23
Heard some message	31.30	40.50	23.00	34.77

Source: Ethiopian Demographic and Health Survey (2005 and 2011) data

Table 2 shows that there are gender differentials relating to the socio-economic status of women and men in Ethiopia. The results show that the unemployment status of women has remained the same at 64 % between 2005 and 2011. On the other hand, unemployment among men increased between 2005 and 2011, from 12.40 % to 22 % respectively. The results also show that there is a wide gender gap in employment status. In 2011, just over three in ten women reported that they were employed, whereas over seven in ten men reported that they were employed. Between 2005 and 2011, the employment status of women showed a 0.28 % increase; whereas among employed men there was a 10.96 % decrease between these two periods. The percentage of women in unpaid work has shown a decrease between 2005 and 2011, from 52.50 % to 22.60 % respectively – which is a 56.95 % decline between the two periods. Among men, there has been a 65.76 % decline among those in unpaid work, from 51.40 % in 2005 to 17.60 % in 2011. Women paid in cash or partly in kind increased from 39.70 % in 2005 to 71.10 % in 2011 – with a 79.09 % increase; while among men, those paid in cash or partly in kind showed an increase of over 100 %, from 28.50 % in 2005 to 74.90 % in 2011. Regarding the type of occupation, women and men are mostly employed in agricultural work. The percentage of women employed in agriculture decreased between 2005 and 2011, from 52.20 % to 37.50 % respectively (with a 28.16 % decline). Conversely, the percentage of men in agricultural work has declined by 21.87 % from 2005 to 2011, from 84.60 % to 66.10 % respectively.

**Table 2:** Percentage distribution of socio-economic variables of women empowerment in Ethiopia in 2005 and 2011

Characteristics	DHS 2005		DHS 2011	
	Women	Men	Women	Men
Employment Status				
Unemployed	64.10	12.40	64.00	22.00
Employed	35.90	87.60	36.00	78.00
Type of earning for work				
Unpaid	52.50	51.40	22.60	17.60
Cash only or partly in kind	39.70	28.50	71.10	74.90
Any kind only	7.80	20.10	6.40	7.40
Type of Occupation				
Prof/Tech/Manag.	3.80	2.60	4.30	6.56
Clerical, Sales and services	32.90	7.00	42.00	15.60
Agriculture	52.20	84.6	37.50	66.10
Skilled Manual	6.10	3.30	14.50	9.62
Unskilled manual	5.00	2.50	1.76	2.08

Source: Ethiopian Demographic and Health Survey (2005 and 2011) data

Table 3 shows that there has been an increase in the percentage of women never married between 2005 and 2011. Women who have never married have increased by 20.80 % between 2005 and 2011, from 25.00 % to 30.20 % respectively. Currently married and ever married men have declined from 56.80 % to 55.45 % and 3.10 % to 2.90 % respectively between 2005 and 2011. The fertility preference of women wanting another child or undecided has improved between 2005 and 2011, from 61.40 % to 72.00 %. But among women who do not want any more children the percentage has fallen from 38.60 % to 28.00 % between these two periods. On the other hand, fertility preferences among men regarding those who want another child or are undecided the percentage has fallen from 80.60 % to 71.38 %; and among men who do not want any more children the percentage has increased between 2005 and 2011, from 19.40 % to 28.62 %. The highest percentage of women’s ideal number of children in 2005 was 3 or 4 (32 %), followed by 5 or 6 (20 %), and 7+ at about 16 %. In 2011 these percentages has proved to be a different and the order (from highest to lowest as in 2005) changed, the highest was 3 or 4 children ( 34 %), followed by 7+ at about 19 %, and 5 or 6 children at around 17 %. On the other hand, among men – the highest percentage in 2005 at 3 or 4 children (37 %), followed by 5 or 6 children (21 %) and 7+ at 19 %. From 2005 to 2011 the women's knowledge of modern contraceptive methods increased from 86 % to 94 %.

**Table 3:** Percentage distributions of gender differentials in reproductive roles based on background characteristics in Ethiopia, DHS 2005 and 2011

Background characteristics	(Model I) DHS 2005		(Model II) DHS 2011	
	Proportion of Women	Proportion of Men	Proportion of Women	Proportion of Men
Marital status				
Never married	25.00	40.10	30.20	41.64
Currently married	64.40	56.80	64.85	55.45
Ever married	10.60	3.10	5.00	2.90
Fertility preference (desire for more children)				
Want another or undecided	61.40	80.60	72.00	71.38
Want no more	38.60	19.40	28.00	28.62
Ideal number of children				
0 child	11.00	2.80	6.40	2.60
1–2 children	11.00	13.30	13.50	14.50
3–4 children	32.20	37.30	34.10	35.80
5–6 children	19.70	20.60	17.00	16.50
7+ children	15.60	19.10	18.70	22.50
Non-numeric response	10.40	6.90	10.40	8.10
Knowledge of family planning method				
Knows no method	13.90	9.00	5.30	2.20
Knows traditional method only	0.10	0.30	0.30	0.10
Knows modern method	86.00	90.70	94.30	97.70

Source: Ethiopian Demographic and Health Survey (2005 and 2011) data

### A multivariate approach

For the purpose of the regression analysis, those who have reported to have primary education and above are referred to as those with some education. In 2011 women aged 25–34 were 0.255 times less likely to attain some education compared to those aged 15–24. However, in 2011 men aged 25–34 were 2.274 times more likely to attain some education than men aged 15–24. This shows that when comparing men to women, men were more educated than women in both2005 and 2011. In both 2005 and 2011 women in the richest wealth quintile had the highest odds ratio relating to some education. In 2011 women and men in the richest quintile were respectively 10.904 and 2.131 times more likely to attain some education compared to the poorest quintile. Women from a protestant religion were 1.703 times more likely to attain some education than those of the orthodox religion in 2005. In both 2005 and 2011, women in Addis Ababa had the highest odds ratios relating to some education, with corresponding odds ratios of 1.53 (2005) and 6.4 (2011). This indicates that women in Addis Ababa were about 1.53 (in 2005) and 6.4 (in 2011) times more likely to attain some education than those in Tigray region (Table 4).

**Table 4:** Multivariate model (odds ratios) showing factors associated with married women’s empowerment by two dependent variables (education and work status)

Variables	Dependent variable: Education (with primary education and above =1 and illiterate = 0)				Dependent variable: Work Status (Yes = 1 and No = 0)			
	DHS 2005 (N = 8914)		DHS 2011 (N = 9594 )		DHS 2005 (N = 8914)		DHS 2011 (N = 9594)	
	Women	Men	Women	Men	Women	Men	Women	Men
	Model I EXP(β)	Model II EXP(β)	Model I EXP(β)	Model II EXP(β)	Model I EXP(β)	Model II EXP(β)	Model I EXP(β)	Model II EXP(β)
*Age of respondent*		***	***			***	***	
15–24	-	-	-					
25–34	0.68	0.78	0.26	2.27	1.35	5.75	1.60	2.60
35–44	0.30	0.53	0.17	1.71	1.60	2.88	1.48	2.96
45–49	0.11	0.20	0.09	3.23	1.39	1.49	1.46	2.86
*Wealth quintile*			***	***				
Poorest								
Poorer	1.17	1.65	1.51	1.20	1.07	1.22	1.27	1.21
Middle	1.87	2.33	1.81	1.27	1.03	1.97	1.58	1.10
Richer	2.70	4.21	2.79	3.42	1.19	1.77	1.68	1.04
Richest	6.39	10.33	10.90	2.13	1.25	1.62	2.89	0.85
*Place of residence*								
Urban								
Rural	3.89	2.58	6.44	0.12	1.15	0.06	2.19	1.36
*Region*			***	***			***	
Tigray								
Affar	0.38	0.51	0.27	0.55.	0.13	0.68	0.46	0.39
Amhara	0.65	0.45	0.58	0.93	0.61	0.52	1.01	0.52
Oromiya	1.11	1.76	1.09	1.13	0.70	0.69	1.65	1.40
Somali	0.19	0.33	0.29	0.52	0.15	0.75	0.56	0.57
Ben-Gumz	0.92	0.89	0.73	1.35	1.01	1.29	1.82	0.90
SNNP	0.70	1.51	1.09	1.57	0.44	0.67	1.64	1.82
Gambela	1.27	1.98	1.65	0.96	0.64	1.15	1.56	2.47
Harari	1.21	1.62	1.86	0.36	0.64	2.75	1.58	2.94
Addis Ababa	1.53	1.59	6.37	1.45	0.56	1.13	2.72	12.02
Dire Dawa	0.85	1.59	1.46	1.01	0.43	1.73	1.51	1.89
*Religion*			***	***			***	
Orthodox								
Protestant	1.70	1.54	0.97	1.92	0.76	0.63	0.99	1.05
Muslim	0.55	0.62	0.38	0.60	0.80	0.43	0.54	0.72
Traditional	0.59	0.88	0.24	0.36	1.36	????	1.08	3.68
Catholic and Others	1.25	1.39	0.49	0.93	1.00	0.48	1.24	1.18

Source: Ethiopian Demographic and Health Survey (2005 and 2011) data

Therefore, according to the different regions, the highest odds ratios for women with some education was recorded in Addis Ababa in 2011. But, when it comes to the highest odds ratios among men who had some education in 2005, then men within the Gambela region had the highest odds ratios (of about 2) in 2005, whereas in 2011 the SNNP region had the highest odds ratios among men at about 1.6. This shows that in 2005 men in the Gambela region were about 2 times more likely to attain some education than those in the Tigray region. In the SNNP region in 2011 men were about 1.6 times more likely to attain some education than those in the Tigray region.

The highest odds ratios of women who are working shows that in 2005 it was 1.599 and in 2011 the highest odds ratios was 1.57. These results show that in 2005 women aged 35–44 were 1.599 times more likely to be in work than women aged 15–24. In 2011, women aged 25–34 were 1.57 times more likely to be working than those aged 15–24. Moreover, this shows that when comparing 2005 and 2011, women were in employment at younger ages (i.e. aged less than 35 years) in 2011 than in 2005. Whereas, in 2011 it was mostly men aged 35 and above who were working compared to those younger the 35 years. In 2005 (OR = 1.25) and 2011 (OR = 2.89), the richest quintile had the highest odds ratios of women who were engaged in some work. Thus, when comparing the two periods, the richest wealth quintile showed an increase in the odds ratios of working women from 2005 to 2011.

This indicates that in 2011, richest women were about 3 times more likely to be engaged in work compared to poorest women. In 2005, the middle wealth quintile was the highest among men who reported to be in work, at about 1.967. These indicate that men from the middle wealth quintile were 1.967 times more likely to be engaged in work than those in the poorest wealth quintile. Concerning religion, the traditional religion had the highest odds ratios of women who reported to be working in 2005 at about 1.363. But the religion categorized as Catholic and others had the highest odds ratios of women who were working in 2011 at about 1.24. This indicates that women from the traditional religion were 1.363 times more likely to be in work than those in the orthodox religion in 2005, whereas in 2011 women from the catholic and other religion were 1.24 times more likely to be engaged in work than those in orthodox religion. The finding show that men from the protestant religion were 0.625 times less likely to be in work than those of men from orthodox religion in 2005, whereas in 2011 men from the traditional religion were 3.683 times more likely to be engaged in work than those in the orthodox religion. In 2005 and 2011, the highest odds ratios of women who were engaged in work was that of women from Ben-Gumz (about 1.006) and Addis Ababa (about 12.02) compared to Tigray. However, the highest odds ratios of men who were working between these two periods are among men from Harari (at about 2.747 in 2005) and Addis Ababa (at about 12.02 in 2011) (Table 4).

## Discussion

 In both 2005 and 2011, it had been mostly women who had no educational attainment. In 2011, 50 % of women had no education compared with 32 % of men. The results clearly shows the gender gap in education in the country. Education has already been related to women’s empowerment; hence it is important to improve women’s education as a way of empowering them and giving them autonomy to have the power to make crucial decisions about their lives. Results from a study conducted in 2009 -2010 in Ethiopia pointed out that education is among the key factors in women’s empowerment – where it was correlated with knowledge of marriage as well as the autonomy (or power) to engage in discussions about fertility and reproductive health issues in young girls [13]. The multivariate analysis showed that primary and secondary educations are associated with age, wealth quintile, region and religion. Employment of women was significantly less than that of men in both 2005 and 2011. Women who reside in rural areas and those who are from the richest households (characterized by the richest wealth quintile), are more likely to have primary education and above as well as being more likely to be working. In rural areas where education is not encouraged among girls and where there are strict gender roles (i.e. a woman only exists for purposes of marriage and reproduction) – then in such situations tradition and culture do not support women to go out and work for an income. 

As the result of the negative effect of culture and tradition, husbands do not permit women to go out and work; if not they will be considered as turning aside from the norm. Besides this cultural obstruction, there seems to be further economic restriction among the poor uneducated women where these restrictions make it difficult for them to start even small scale income-producing work. In many cases, having many children, coupled with the heavy daily workload at home to maintain the family, does not leave much time to venture working outside. The cultural barriers preventing women from working to earn a living were strongly stated in Gambella, Somali and SNNP regions. The results indicated that men have shown greater desire for having more children than women in 2005. In 2011, this trend changed, where women and men had almost the same desire for having more children which was 72 % and 71.4 % respectively. The demand for limiting family size was higher for women than men in 2005. Conversely, in 2011 the demand for limiting family size was higher for men than women. On the other hand, men are the principal if not the sole decision makers regarding controlling fertility of women in most of the societies particularly in rural areas. Health seeking behaviour of women is lacking, we need to promote women empowerment and women decision making capacity/power through education. A study on gender inequality and the empowerment of women in Ethiopia suggested that children are often held in high regard in the country, where they are seen as prised-possessions who will grow up and benefit the parents financially (especially girls who will be married off and thus bring wealth, through dowry, to the family) [5]. It is this kind of thinking that serves as the barrier to women empowerment – especially where young girls are regarded as having less value than boys. Culture and religion are often used interchangeably to promote or justify the worldviews of those who practice customs such as child marriages [5].

## Conclusion

The women’s empowerment namely education and employment status, are positively associated with health, wealth index and age of respondent, region, and religion. In addition, the study indicated that high gender disparity on socio-economic indicators have been recorded in Ethiopia – where men tend to fair higher than women. We conclude that married women with educational attainment of primary education and above are less likely get married at an early age than those who are uneducated. This does not eliminate the chances of being married at an early age (i.e. at ages less than 18 years) but it suggests that it limits and empowers girls to be aware of their rights. Thus, we recommend that women must be empowered in terms of decision making power, purchasing power, special policy to promote female education, health care, rise birth interval minimum 24 months, increasing age at marriage and be integrated into family planning programming in Ethiopia; and that these issues must be focused at the vulnerable group (which is young girls, those aged less than 18 and those aged 18–24). Rural mothers community participation, health care service providers, policy makers, human resources, program managers, government and non-government organizations must work together to eliminate gender inequality and provide equal status to men and women. There was significant relationship between status of women and public health, and this relationship appeared to differ by education and work status. Further in depth qualitative analysis is recommended.

## Consent

Consent was not necessary because the study used secondary data from the Ethiopian Demographic and Health Survey 2005 and 2011 Email: info@measuredhs.com, Internet: (http://www.measuredhs.com) All data were deidentified. If additional information about the data may be obtained from the Central Statistical Agency, Email: csa@ethionet.et.

## Abbreviations

EDHS: Ethiopian Demographic and Health Survey
SNNP: Southern Nations, Nationalities, and Peoples’ Region

## References

[1] Centre W’s I (2005). Training Manual for Gender Planning.

[2] Hirut T, Chowdhury S, Wais A, Kahsai Wolde G (2004). Violence Against Women in Ethiopia: A Strong Case of Civil Society Concern. Civil Society in Ethiopia: Reflections on Realities and Perspectives of Hope. African – Asian Studies Promotion Association.

[3] Kabira WM, Masinjila M (1997). ABC of gender analysis.

[4] Mukuria A, Casey A, Albert T (2005). The Context of Women’s Health: Results from the Demographic and Health Surveys, 1994–2001. Comparative Reports No. 11.

[5] Ethiopian Society of Population Studies (2008). Gender Inequality and Women’s Empowerment: In-depth Analysis of the Ethiopian Demographic and Health Survey, 2005.

[6] Kishor S (2005). A Focus on Gender: Collected Papers on Gender Using DHS Data.

[7] Kishor S, Presser HB, Sen G (2000). Empowerment of women in Egypt and links to the survival and health of their infants. Women’s empowerment and demographic processes: Moving beyond Cairo.

[8] Haregewoin C, Emebet M (2003). Towards Gender equality in Ethiopia, A Profile of Gender Relations.

[9] Santhya KG, Haberland N, Das A, Lakhani A, Ram F, Sinha RK (2008). Empowering married young women and improving their sexual and reproductive health: effects of the First-time Parents Project.

[10] Ogato GS (2013). The quest for gender equality and women’s empowerment in least developed countries: Policy and strategy implications for achieving millennium development goals in Ethiopia. International Journal of Sociology and Anthropology;.

[11] Wado YD (2013). Women’s Autonomy and Reproductive Healthcare-Seeking Behavior in Ethiopia. DHS working papers No. 91.

[12] Darteh EKM, Doku DT, Esia-Donkoh K (2014). Reproductive health decision making among Ghanaian women. Reprod Health.

[13] Erulkar A (2013). Early marriage, marital relations and intimate partner violence in Ethiopia. Int Perspect Sex Reprod Health.

